# Non-Hermitian Floquet Phases with Even-Integer Topological Invariants in a Periodically Quenched Two-Leg Ladder

**DOI:** 10.3390/e22070746

**Published:** 2020-07-07

**Authors:** Longwen Zhou

**Affiliations:** Department of Physics, College of Information Science and Engineering, Ocean University of China, Qingdao 266100, China; zhoulw13@u.nus.edu

**Keywords:** non-hermitian system, floquet system, topological phase, dynamics

## Abstract

Periodically driven non-Hermitian systems could possess exotic nonequilibrium phases with unique topological, dynamical, and transport properties. In this work, we introduce an experimentally realizable two-leg ladder model subjecting to both time-periodic quenches and non-Hermitian effects, which belongs to an extended CII symmetry class. Due to the interplay between drivings and nonreciprocity, rich non-Hermitian Floquet topological phases emerge in the system, with each of them characterized by a pair of even-integer topological invariants $(w_{0},w_{\pi })\in 2Z\times 2Z$. Under the open boundary condition, these invariants further predict the number of zero- and π-quasienergy modes localized around the edges of the system. We finally construct a generalized version of the mean chiral displacement, which could be employed as a dynamical probe to the topological invariants of non-Hermitian Floquet phases in the CII symmetry class. Our work thus introduces a new type of non-Hermitian Floquet topological matter, and further reveals the richness of topology and dynamics in driven open systems.

## 1. Introduction

Non-Hermitian states of matter have attracted great attention in recent years due to their intriguing dynamical and topological properties (see [1,2,3,4,5,6,7,8] for reviews). Theoretically, a wide range of non-Hermitian topological phases and phenomena have been classified and characterized according to their symmetries [9,10,11,12,13,14,15,16,17] and dynamical signatures [18,19,20,21,22,23]. Experimentally, non-Hermitian topological matter have also been realized in cold atom [24,25], photonic [26,27,28,29], acoustic [30,31,32], electrical circuit [33,34,35] systems, and nitrogen-vacancy-center in diamond [36], leading to potential applications such as topological lasers [37,38,39] and high-performance sensors [40,41,42,43]. Additionally, non-Hermitian structures could also arise in supersymmetric quantum mechanics, where a series of supersymmetric models have been solved exactly [44,45,46,47,48].

Recently, the study of non-Hermitian physics has been extended to Floquet systems, in which the interplay between time-periodic driving fields and gains/losses or nonreciprocal effects could potentially yield topological phases that are unique to driven non-Hermitian systems [49,50,51,52,53,54,55,56,57,58,59,60,61,62,63]. In early studies, various non-Hermitian Floquet topological phases and phenomena have been discovered, including non-Hermitian Floquet topological insulators [49,50,53,54,55], superconductors [52], semimetals [63], and skin effects [56,57]. Meanwhile, the time-averaged spin texture and mean chiral displacement have been suggested as two dynamical tools to extract the topological invariants of non-Hermitian Floquet systems [49,50,51,53]. These discoveries extend the boundary of nonequilibrium phases of matter to driven non-Hermitian systems, and shed light on new approaches for the detection of their intriguing features.

In previous studies, non-Hermitian Floquet phases were explored mainly in two-band systems. According to the periodic table of topological matter, the CII symmetry class refers to the chiral symplectic class. A topological phase in this symmetry class is protected by the time-reversal, particle-hole, and chiral symmetries [64,65]. Moreover, the tight-binding Hamiltonian describing a system in the CII symmetry class possesses a minimum number of four bands. In this work, we uncover a new type of non-Hermitian Floquet topological matter in the extended CII symmetry class, which contains at least four quasienergy bands. Our system can be realized in a periodically quenched nonreciprocal two-leg ladder, as introduced in Section 2. Each topological phase of the system is characterized by a pair of even-integer winding numbers $(w_{0},w_{\pi })\in 2Z\times 2Z$. With the change of the nonreciprocal parameters of the model, we find rich non-Hermitian Floquet phases with large winding numbers, and various topological phase transitions induced by non-Hermitian effects, as presented in Section 3. In Section 4, we obtain multiple quartets of non-Hermitian Floquet edge modes in our system at zero and π quasienergies under the open boundary condition (OBC), and establish the correspondence between the number of these modes and bulk topological invariants $\left(w_{0},w_{\pi }\right)$. In Section 5, we explore the dynamical aspects of our model by generalizing the mean chiral displacement (MCD) to non-Hermitian Floquet systems in the CII symmetry class, and demonstrate the relationship between the MCDs and topological winding numbers $\left(w_{0},w_{\pi }\right)$. Finally, we summarize our findings and discuss potential future directions in Section 6.

## 2. Model and Symmetry

The model we are going to investigate can be viewed as a driven, non-Hermitian version of the Creutz ladder (CL) with spin-1/2 fermions and spin-orbit couplings (or spinless particles with four sublattice degrees of freedom). The CL model refers to a quasi-one-dimensional lattice formed by two coupled legs and subjected to a perpendicular magnetic flux [66]. It could possess symmetry-protected degenerate zero modes at its boundaries, and therefore belong to one of the earliest examples of a topological insulator [66]. In later studies, the CL model has been realized in photonic [67,68] and cold atom [69,70] systems, and utilized in the investigations of Aharonov–Bohm cages [71,72], topological pumping [73], localization [74,75], and many-body topological matter [76,77,78,79,80]. Recently, spin-1/2 extensions of the CL model have also been explored in several studies [81,82,83], leading to the discoveries of richer topological features. Furthermore, when time-periodic drivings are applied to the spin-1/2 CL, a series of Hermitian Floquet topological phases in the CII symmetry class were found [84]. Each of these phases is characterized by a pair of even-integer topological winding numbers, quantized dynamics of bulk states, together with degenerate quartets of zero and π Floquet edge modes under the OBC [84]. In this work, the construction of our system can be viewed as a non-Hermitian extension of the model studied in Ref. [84], and will be referred to as the non-Hermitian periodically quenched two-leg ladder (PQTLL).

The time-dependent Hamiltonian of the non-Hermitian PQTLL model takes the form:

(1) $$H\left(t\right)=\left\{\begin{array}{cc} H_{\|} & t\in [jT,jT+T/2)\\ H_{⊥} & t\in [jT+T/2,jT+T) \end{array}\right.,$$

where j∈Z and *T* is the driving period. Within the first (second) half of each driving period, the system is described by the time independent Hamiltonian $H_{\|}$ ($H_{⊥}$). In the middle of the driving period, a sudden quench is applied to the system, so that its Hamiltonian is switched from $H_{\|}$ to $H_{⊥}$. Throughout this work, we will set ℏ=T=1 as the convention of units. In the lattice representation, the Hamiltonian components $H_{\|}$ and $H_{⊥}$ are explicitly given by:

(2) $$\begin{array}{cc} H_{\|} & =\sum_{n}J_{x}(|n\rangle \langle n+1|+H.c.)\sigma_{0}⊗\tau_{z}-\sum_{n}iV(|n\rangle \langle n+1|-H.c.)\sigma_{y}⊗\tau_{0}, \end{array}$$

(3) $$\begin{array}{cc} H_{⊥} & =\sum_{n}J_{y}\left|n\rangle \langle n\right|\sigma_{0}⊗\tau_{x}+\sum_{n}iJ_{d}(|n\rangle \langle n+1|-H.c.)\sigma_{z}⊗\tau_{x}. \end{array}$$

**Figure 1:**
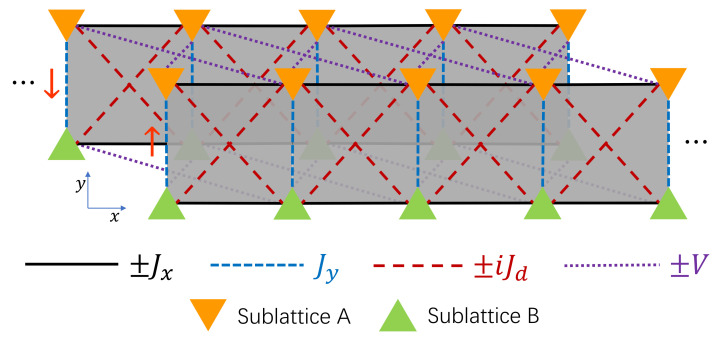
Schematic diagram of the non-Hermitian two-leg ladder model. The forward (backward) copy of the ladder corresponds to the spin up (down) components. Each unit cell of the ladder contains two sublattices A and B. The intracell and intercell coupling parameters are denoted explicitly in the figure. In the first (second) half of a driving period, only the couplings $\left(J_{x},V\right)$ [$\left(J_{y},J_{d}\right)$] are switched on.

Here n=1,…,N are the indices of unit cells, which are arranged along the horizontal (x) direction of the ladder. $\sigma_{0}$ and $\tau_{0}$ are both 2×2 identity matrices. An illustration of the model is presented in Figure 1. Each unit cell of the ladder contains two spin and sublattice components, and $\sigma_{x,y,z}$, $\tau_{x,y,z}$ are Pauli matrices acting on the spin-1/2 and sublattice degrees of freedom, respectively. The system parameters $J_{x}$ and $J_{y}$ represent the intercell and intracell hopping amplitudes along the horizontal (*x*) and vertical (*y*) directions of the ladder. $J_{d}$ controls the coupling strength between different sublattices in adjacent unit cells, and *V* describes the amplitude of spin-orbit coupling among particles with opposite spins in the same sublattice of nearest-neighbor unit cells. In this work, we allow $J_{y}$ and $J_{d}$ to take complex values, i.e., $J_{y}=J_{y}^{r}+iJ_{y}^{i}$ and $J_{d}=J_{d}^{r}+iJ_{d}^{i}$. Physically, the imaginary parts $J_{y}^{i}$ and $J_{d}^{i}$ could describe the nonreciprocity of hoppings along the vertical and diagonal directions of the ladder.

The Floquet operator of the non-Hermitian PQTLL model, which corresponds to its evolution operator over a complete driving period (e.g., from $t=j+0^{-}$ to $j+1+0^{-}$), can be expressed as:

(4) $$U=Te^{-i\int_{0}^{1}H\left(t\right)dt}=e^{-\frac{i}{2}H_{⊥}}e^{-\frac{i}{2}H_{\|}},$$

where T is the time-ordering operator, which directly leads to the last equality as the system Hamiltonian in Equation (1) is time-independent within each half of the driving period. The quasienergy spectrum ε of the system can be obtained by solving the eigenvalue equation $U|\psi \rangle =e^{-i\varepsilon }|\psi \rangle$ under a fixed boundary condition, where |ψ〉 is the corresponding right eigenvector of *U*. With a ladder of *N* unit cells and under the period boundary condition (PBC), one can perform the Fourier transform $|n\rangle =\frac{1}{\sqrt{N}}\sum_{k}e^{-ink}|k\rangle$ to express *U* in momentum space as $U=\sum_{k}\left|k\rangle U\left(k\right)\langle k\right|$. Here k∈[−π,π) is the quasimomentum, and

(5) $$\begin{array}{cc} U(k) & =e^{-ih_{⊥}\left(k\right)}e^{-ih_{\|}\left(k\right)}, \end{array}$$

(6) $$\begin{array}{cc} h_{\|}\left(k\right) & =J_{x}\cos k\sigma_{0}⊗\tau_{z}+V\sin k\sigma_{y}⊗\tau_{0}, \end{array}$$

(7) $$\begin{array}{cc} h_{⊥}\left(k\right) & =\frac{J_{y}}{2}\sigma_{0}⊗\tau_{x}-J_{d}\sin k\sigma_{z}⊗\tau_{y}. \end{array}$$

Since $J_{y}$ and $J_{d}$ are in general complex parameters of the system, U(k) is not unitary. In terms of the biorthogonal eigenbasis of U(k), Equation (5) can be equivalently written as:

(8) $$U\left(k\right)=\sum_{\ell =1,2}\sum_{\eta =\pm }e^{-i\varepsilon_{\ell }^{\eta }\left(k\right)}|\varepsilon_{\ell }^{\eta }\left(k\right)\rangle \langle {\bar{\varepsilon }}_{\ell }^{\eta }\left(k\right)|,$$

where $|\varepsilon_{\ell }^{\eta }\left(k\right)\rangle$ ($\langle {\bar{\varepsilon }}_{\ell }^{\eta }\left(k\right)|$) is the right (left) eigenvector of U(k) with the quasienergy $\varepsilon_{\ell }^{\eta }\left(k\right)=\eta \varepsilon_{\ell }\left(k\right)\in C$. ℓ=1,2 are the indices of the two quasienergy bands, whose real parts satisfy Re[εℓ(k)]∈(0,π]. The complex dispersion $\left\{\varepsilon_{\ell }^{\eta }\left(k\right)\right\}$ thus contains four Floquet bands, with two possible spectral gaps at quasienergies zero and π. A topological phase transition may happen when a gap closes at one of these quasienergies. This can be further captured by the vanishing of one of the two gap functions $\Delta_{0}$ and $\Delta_{\pi }$, defined as:

(9) Δ0≡mink,ℓ[Reεℓ(k)]2+[Imεℓ(k)]2,

(10) Δπ≡mink,ℓ[|Reεℓ(k)|−π]2+[Imεℓ(k)]2.

In the next section, these functions will be utilized to obtain the boundaries between different Floquet topological phases of the non-Hermitian PQTLL model.

The topological invariants of the non-Hermitian Floquet phases in our system are determined by the symmetries of U(k). Following the usual strategy in the study of one-dimensional (1D) Floquet systems [85,86], we rewrite U(k) in a pair of symmetric time frames as:

(11) $$\begin{array}{cc} U_{1}\left(k\right)= & e^{-\frac{i}{2}h_{\|}\left(k\right)}e^{-ih_{⊥}\left(k\right)}e^{-\frac{i}{2}h_{\|}\left(k\right)}=e^{-ih_{1}\left(k\right)}, \end{array}$$

(12) $$\begin{array}{cc} U_{2}\left(k\right)= & e^{-\frac{i}{2}h_{⊥}\left(k\right)}e^{-ih_{\|}\left(k\right)}e^{-\frac{i}{2}h_{⊥}\left(k\right)}=e^{-ih_{2}\left(k\right)}. \end{array}$$

It is clear that $U_{1,2}\left(k\right)$ and U(k) are related by similarity transformations, which can be achieved by shifting the initial time of the driving forward or backward over half a period. The Floquet operators $U_{1,2}\left(k\right)$ thus share the same quasienergy dispersion with U(k), and they can be expressed in their corresponding biorthogonal basis and as:

(13) $$U_{\alpha }\left(k\right)=\sum_{\ell =1,2}\sum_{\eta =\pm }e^{-i\varepsilon_{\ell }^{\eta }\left(k\right)}|\varepsilon_{\alpha \ell }^{\eta }\left(k\right)\rangle \langle {\bar{\varepsilon }}_{\alpha \ell }^{\eta }\left(k\right)|,$$

where α=1,2 denote the two time frames. Moreover, the effective Hamiltonians $h_{1,2}\left(k\right)$ in Equations (11) and (12) both possess the extended time-reversal symmetry T, the extended particle-hole symmetry C, and the sublattice (chiral) symmetry S, i.e.,

(14) $$T=i\sigma_{y}⊗\tau_{0},\;TT^{*}=-1,\;Th_{\alpha }^{⊤}\left(k\right)T^{-1}=h_{\alpha }\left(-k\right),$$

(15) $$C=\sigma_{x}⊗\tau_{y},\;CC^{*}=-1,\;Ch_{\alpha }^{⊤}\left(k\right)C^{-1}=-h_{\alpha }\left(-k\right),$$

(16) $$S=\sigma_{z}⊗\tau_{y},\;S^{2}=1,\;Sh_{\alpha }\left(k\right)S=-h_{\alpha }\left(k\right).$$

According to the symmetry classification of Floquet systems [87] and the periodic table of non-Hermitian topological phases [9,11], the non-Hermitian PQTLL model belongs to an extended CII symmetry class with even-integer topological invariants. In the meantime, the system also possesses the inversion symmetry $P=\sigma_{x}⊗\tau_{0}$ with $P^{2}=1$, in the sense that $Ph_{\alpha }\left(k\right)P^{-1}=h_{\alpha }\left(-k\right)$ for α=1,2. According to Ref. [9], the coexistence of time-reversal and inversion symmetries allows a system to be immune to the non-Hermitian skin effect [88,89,90]. The topological characterization and bulk-boundary correspondence of our non-Hermitian PQTLL model can thus be treated in a standard manner, as will be presented in the following sections. Note in passing that under the combined action of P and T, we have $\left(\mathrm{PT}\right)h_{\alpha }^{⊤}\left(k\right){\left(\mathrm{PT}\right)}^{-1}=h_{\alpha }\left(k\right)$. This is a different form of the PT-symmetry, which in general cannot guarantee the realness of the quasienergy spectrum of $U_{\alpha }\left(k\right)$. Besides, even for a PT-symmetric system, the spectrum can only be real in the PT-invariant regime, and will in general be transformed from real to complex when the system parameters are varied across a PT-symmetry breaking transition [91,92]. Neverless, as will be shown in Section 4, the topological edge modes in our system always possess real quasienergies zero or π. These Floquet edge modes are thus immune to any possible PT-breaking transitions so long as the sublattice symmetry S is preserved.

## 3. Topological Invariants and Phase Diagrams

In this section, we introduce the topological invariants of our non-Hermitian PQTLL model, and construct its topological phase diagrams for typical situations.

Following the symmetry analysis in the last section and the topological characterizations of Hermitian Floquet phases [84,93], the Floquet operator $U_{\alpha }\left(k\right)$ in the α’s time frame possesses a topological winding number $w_{\alpha }$, which can be defined as:

(17) $$w_{\alpha }=\int_{-\pi }^{\pi }\frac{dk}{4\pi }\mathrm{Tr}\left[SQ_{\alpha }\left(k\right)i\partial_{k}Q_{\alpha }\left(k\right)\right],$$

where α=1,2, *k* is the quasimomentum, S is the sublattice symmetry operator, and the trace is taken over all the internal degrees of freedom including spins and sublattices. $Q_{\alpha }\left(k\right)$ is usually called the Q-matrix [94], which takes the form of a biorthogonal projector:

(18) $$Q_{\alpha }\left(k\right)=\sum_{\ell ,\eta }\eta |\varepsilon_{\alpha \ell }^{\eta }\left(k\right)\rangle \langle {\bar{\varepsilon }}_{\alpha \ell }^{\eta }\left(k\right)|.$$

Here ℓ=1,2 are the indices of the two Floquet quasienergy bands, whose real parts are positive. η=± denote the signs of the real parts of quasienergies. The right (left) eigenvectors $\left\{|\varepsilon_{\alpha \ell }^{\eta }\left(k\right)\rangle \right\}$ ($\left\{|{\bar{\varepsilon }}_{\alpha \ell }^{\eta }\left(k\right)\rangle \right\}$) can be obtained by expressing the Floquet operator in the α’s time frame as $U_{\alpha }\left(k\right)=V_{\alpha }\left(k\right)\Lambda_{\alpha }\left(k\right)V_{\alpha }^{-1}\left(k\right)$, where $\Lambda_{\alpha }\left(k\right)$ is diagonal and $\left\{|\varepsilon_{\alpha \ell }^{\eta }\left(k\right)\rangle \right\}$ ($\left\{|{\bar{\varepsilon }}_{\alpha \ell }^{\eta }\left(k\right)\rangle \right\}$) are given by the columns of $V_{\alpha }\left(k\right)$ (${\left[V_{\alpha }^{-1}\left(k\right)\right]}^{†}$) [94]. Therefore, $Q_{\alpha }\left(k\right)$ can be viewed as a flattened effective Hamiltonian of $U_{\alpha }\left(k\right)$, whose eigenphases with positive and negative real parts are set to zero and π, respectively. Note that since $Q_{\alpha }$ is given by the difference between the projectors of two sets of bulk quasienergy bands (with η=±), its formalism does not rely on the exact number of bands possessed by the system [9]. Indeed, through the formalism of the projector in Equation (18), only the net information of bands contained in the two quasienergy ranges Re(ε)∈(−π,0) and Re(ε)∈(0,π) are taken into accout, which generalizes the winding number of two-band systems to multiple-band cases [9]. For a system with the sublattice symmetry S and the spectral gaps at ε=0,π, the total number of bulk bands is even. Furthermore, the set of Pauli matrices $\sigma_{x,y,z}$ plus the 2×2 identity $\sigma_{0}$ could not satisfy all the symmetry requirements of the CII class (i.e., ${\mathrm{TT}}^{*}=-1$, ${\mathrm{CC}}^{*}=-1$ and $S^{2}=1$) simultaneously. Therefore, a 1D Floquet system in the CII symmetry class contains at least four quasienergy bands. The model we introduced satisfies this requirement for the minimal number of bulk bands.

With the help of $\left(w_{1},w_{2}\right)$ in Equation (17) and the topological characterization of chiral symmetric Floquet systems [86], we can construct another pair of topological winding numbers $\left(w_{0},w_{\pi }\right)$ as:

(19) $$w_{0}=\frac{w_{1}+w_{2}}{2},\;w_{\pi }=\frac{w_{1}-w_{2}}{2}.$$

According to Ref. [84], these invariants would only take even-integer values, and they provide a complete characterization for all 1D Hermitian Floquet topological phases in the CII symmetry class. Furthermore, the requirement of two invariants reveals the difference between Floquet and non-driven systems. Since the Floquet operator *U* possesses two quasienergy gaps at ε=0 and π, there could be two types of degenerate edge modes at these quasienergies, whose numbers are thus counted by two distinct topological invariants. In the following, we will demonstrate that the topological invariants $\left(w_{0},w_{\pi }\right)$ in Equation (19) could also characterize the Floquet phases of the non-Hermitian PQTLL model, and they always take real and even-integer values for a gapped topological phase. Note in passing that for our system, the spectral gaps take the form of lines through zero and π quasienergies. For a non-driven system with the sublattice symmetry S, the non-Hermitian topological phases can be characterized by a winding number w∈2Z [9], which count the number of zero-energy edge modes under the open boundary conditions. Our results extend this topological characterization to 2Z×2Z, with the second even integer $w_{\pi }$ being related to the line-gap induced at the quasienergy π by the periodic driving fields.

**Figure 2:**
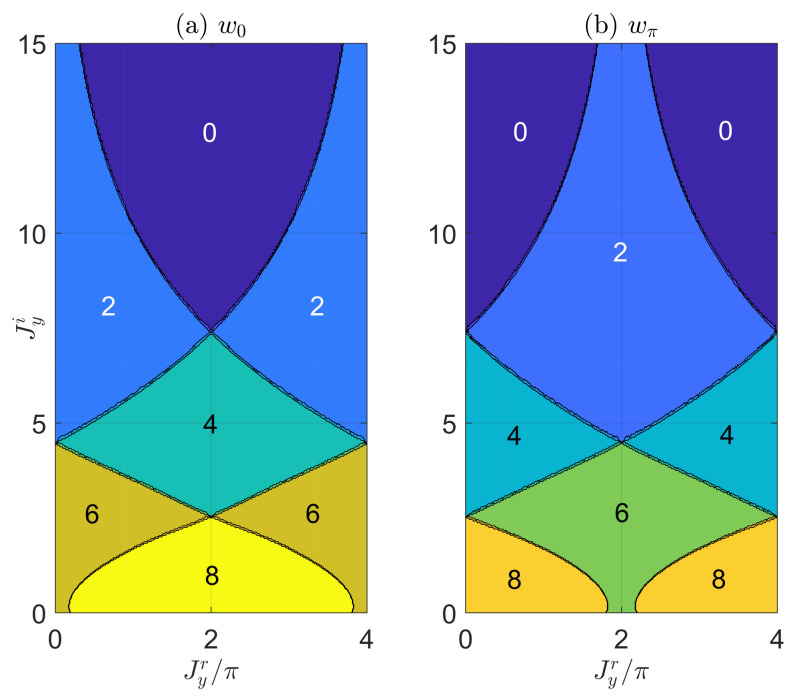
The topological winding numbers $w_{0}$ [in panel (**a**)] and $w_{\pi }$ [in panel (**b**)] versus the real and imaginary parts of the vertical hopping amplitude $J_{y}^{r}$ and $J_{y}^{i}$. The other system parameters are chosen as $\left(J_{x},J_{d},V\right)=\left(0.5\pi ,4\pi ,0.1\pi \right)$. In both panels, each region with a uniform color corresponds to a Floquet topological phase of the non-Hermitian periodically quenched two-leg ladder (PQTLL) model, with the values of winding numbers $\left(w_{0},w_{\pi }\right)$ denoted explicitly therein. The lines separating different regions are the boundaries between different topological phases, which can be obtained numerically from the gap closing conditions $\Delta_{0}=0$ and $\Delta_{\pi }=0$ with the help of Equations (9) and (10).

**Figure 3:**
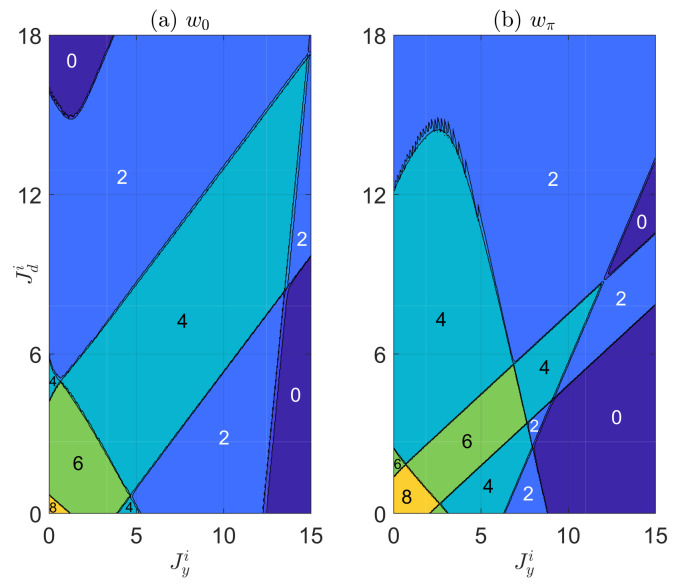
The topological winding numbers $w_{0}$ [in panel (**a**)] and $w_{\pi }$ [in panel (**b**)] versus the imaginary parts of vertical and diagonal hopping amplitudes $J_{y}^{i}$ and $J_{d}^{i}$. The other system parameters are set as $\left(J_{x},J_{y}^{r},J_{d}^{r},V\right)=\left(0.5\pi ,0.6\pi ,4\pi ,0.1\pi \right)$. In both panels, each region with a uniform color refers to a Floquet topological phase of the non-Hermitian PQTLL model, with the values of winding numbers $\left(w_{0},w_{\pi }\right)$ shown explicitly in the figure. The lines separating different regions are the boundaries between different non-Hermitian Floquet topological phases, which can be obtained numerically by setting $\Delta_{0}=0$ and $\Delta_{\pi }=0$ in Equations (9) and (10).

By evaluating $\left(w_{0},w_{\pi }\right)$ numerically with Equations (17) and (18), we obtain the topological phase diagrams of the non-Hermitian PQTLL model for two typical cases, as presented in Figure 2 and Figure 3. In Figure 2, we show the phase diagram of the system with respect to the real and imaginary parts of the vertical hopping amplitude $J_{y}^{r}$ and $J_{y}^{i}$. The other system parameters are all chosen to be real and set as $\left(J_{x},J_{d},V\right)=\left(0.5\pi ,4\pi ,0.1\pi \right)$. From the phase diagram, we see clearly that with the increase of the nonreciprocal parameter $J_{y}^{i}$, a series of topological phase transitions can be induced, with each of them being followed by the quantized change of $w_{0}$ or $w_{\pi }$ by an integer multiple of two. The resulting non-Hermitian Floquet topological phases could possess large and even-integer topological invariants due to the interplay between drivings and non-Hermitian effects. Moreover, phases carrying larger topological winding numbers can be realized when the diagonal hopping amplitude $J_{d}$ takes larger values. Therefore, the realization of non-Hermitian PQTLL model could also provide us with a convenient platform to explore non-Hermitian phases with large and even-integer topological numbers.

In Figure 3, we present the topological phase diagram of the non-Hermitian PQTLL model versus the imaginary parts of the vertical and diagonal hopping amplitudes $J_{y}^{i}$ and $J_{d}^{i}$. The other system parameters are fixed at $\left(J_{x},J_{y}^{r},J_{d}^{r},V\right)=\left(0.5\pi ,0.6\pi ,4\pi ,0.1\pi \right)$. From the phase diagram, we again observe rich non-Hermitian Floquet topological phases characterized by $(w_{0},w_{\pi })\in 2Z\times 2Z$, and multiple topological phase transitions induced by the change of the two non-Hermitian parameters. Furthermore, in certain regions of the phase diagram (e.g., around $J_{y}^{i}=6$), we find phase transitions accompanied by the increase of topological winding numbers $\left(w_{0},w_{\pi }\right)$ when the value of $J_{d}^{i}$ rises.The emergence of such phases with stronger topological signatures in deeper non-Hermitian regimes (here at larger $J_{d}^{i}$) is unique to Floquet non-Hermitian systems. In the meantime, it also suggests an approach to prepare topological phases with large winding numbers under the collaboration of drivings and nonreciprocity.

In the following two sections, we will present the edge states and bulk dynamics of the non-Hermitian PQTLL model, which would provide us with more transparent signatures about its topological properties.

## 4. Edge States and Bulk-Edge Correspondence

One of the key features for a gapped topological phase is the presence of degenerate edge states under the OBC [95]. In this section, we demonstrate the existence of Floquet topological edge modes at zero- and π-quasienergies in our non-Hermitian PQTLL model, and relate their numbers to the bulk topological winding numbers $\left(w_{0},w_{\pi }\right)$ in Equation (19).

The Floquet quasienergy spectrum of our system under the OBC is obtained by solving the quasienergy eigenvalue equation $U|\psi \rangle =e^{-i\varepsilon }|\psi \rangle$, with the Floquet operator *U* given by Equation (4). With the quasienergy ε, we can define the gap functions under the OBC as:

(20) Δ0≡(Reε)2+(Imε)2,Δπ≡(|Reε|−π)2+(Imε)2.

It is clear that $\Delta_{0}=0$ ($\Delta_{\pi }=0$) only when the spectrum gap closes at the quasienergy 0 (π). $\left(\Delta_{0},\Delta_{\pi }\right)$ can thus be used to characterize the behaviors of the Floquet spectrum around the quasienergies ε=0 and π.

**Figure 4:**
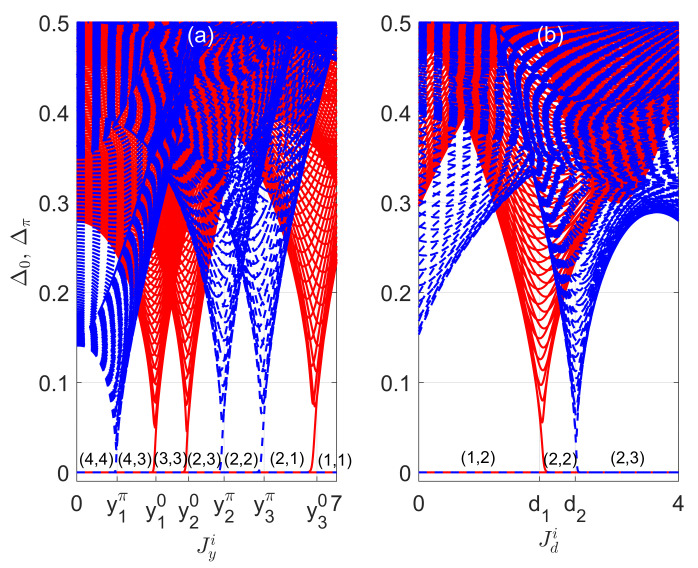
Gap functions $\Delta_{0}$ (red solid lines) and $\Delta_{\pi }$ (blue dashed lines) versus the imaginary part of vertical and diagonal hopping amplitudes $J_{y}^{i}$ and $J_{d}^{i}$ in panels (**a**,**b**), respectively. The system parameters are $\left(J_{x},J_{y}^{r},J_{d},V\right)=\left(0.5\pi ,1.5\pi ,4\pi ,0.1\pi \right)$ for panel (**a**) and $\left(J_{x},J_{y},J_{d}^{r},V\right)=\left(0.5\pi ,0.6\pi +6i,4\pi ,0.1\pi \right)$ for panel (**b**). The number of quartets of zero and π edge modes $\left(n_{0},n_{\pi }\right)$ are denoted explicitly near $\Delta_{0}=\Delta_{\pi }=0$ in both panels, which are related to the winding numbers $\left(w_{0},w_{\pi }\right)$ through the relations in Equation (21). The ticks along the horizontal axis denote the bulk gap closing points, whose numerical values are $\left(y_{1}^{\pi },y_{1}^{0},y_{2}^{0},y_{2}^{\pi },y_{3}^{\pi },y_{3}^{0}\right)\approx \left(1.09,2.14,3.02,3.97,5.05,6.47\right)$ in panel (**a**) and $\left(d_{1},d_{2}\right)\approx \left(1.86,2.41\right)$ in panel (**b**).

In Figure 4a,b, we present the gap functions $\Delta_{0}$ (red solid lines) and $\Delta_{\pi }$ (blue dashed lines) of the non-Hermitian PQTLL model versus the imaginary parts of the vertical and diagonal hopping amplitudes $J_{y}^{i}$ and $J_{d}^{i}$ for two typical sets of system parameters, respectively. In both panels, we see clearly that with the increase of the nonreciprocal hopping parameter $J_{y}^{i}$ or $J_{d}^{i}$, the system undergoes a series of topological phase transitions. Each transition is accompanied by the closing and reopening of a line gap through the quasienergy zero or π, together with the increase or decrease of the number of Floquet zero or π edge modes by an integer multiple of four, as denoted in the figure. Intriguingly, by enhancing the strength of nonreciprocity, we observe transitions from topological phases with smaller winding numbers $\left(w_{0},w_{\pi }\right)$ to larger ones with more edge modes in Figure 4b. The physical mechanism behind this interesting observation is again the interplay between drivings and non-Hermitian effects. Besides, it also indicates the possibility of preparing non-Hermitian Floquet topological states with the help of nonreciprocity.

Furthermore, comparing the number of quartets of the zero (π) edge modes $n_{0}$ ($n_{\pi }$) and the bulk winding number $w_{0}$ ($w_{\pi }$) in each regime of the non-Hermitian Floquet topological phase, we find the following bulk-edge correspondence relations:

(21) $$|w_{0}|=2n_{0},\;\left|w_{\pi }\right|=2n_{\pi }.$$

These relations hold so long as the symmetries that are protecting the non-Hermitian Floquet topological phases of the system are preserved. Experimentally, Equation (21) could also provide us with a window to look into the topological invariants of non-Hermitian Floquet systems in the CII symmetry class by imaging the edge modes. More generally, in the symmetric time frame α (=1,2), we can directly define a noncommutative winding number [84,94,96,97] under the OBC as:

(22) $${\overset{˘}{w}}_{\alpha }\equiv \frac{1}{2N_{B}}{\mathrm{Tr}}_{B}\left(SQ_{\alpha }\left[Q_{\alpha },\hat{n}\right]\right),$$

where S is again the sublattice symmetry operator, and $\hat{n}=\sum_{n=1}^{N}n|n\rangle \langle n|\sigma_{0}⊗\tau_{0}$ is the unit-cell position operator of the ladder. The total number of unit cells *N* contains two parts, i.e., $N=N_{B}+2N_{E}$, where $N_{B}$ and $N_{E}$ are the number of unit cells in the bulk ($n\in [N_{E}+1,N_{E}+N_{B}]$) and edge ($n\in \left[1,N_{E}\right]\cup \left[N-N_{E}+1,N\right]$) regions of the system, and the trace ${\mathrm{Tr}}_{B}\left(\cdot \right)$ is only taken over the bulk degrees of freedom. Different from the previous study [84], the Q-matrix for our non-Hermitian Floquet system in the α’s time frame and under the OBC is expressed in the biorthogonal basis as:

(23) $$Q_{\alpha }\equiv \sum_{n,\eta }\eta |\varepsilon_{\alpha n}^{\eta }\rangle \langle {\bar{\varepsilon }}_{\alpha n}^{\eta }|,$$

where n=1,2,…,2N, η=±, and $|\varepsilon_{\alpha n}^{\eta }\rangle$ is the right Floquet eigenvector satisfying $U_{\alpha }|\varepsilon_{\alpha n}^{\eta }\rangle =e^{-i\varepsilon_{n}^{\eta }}|\varepsilon_{\alpha n}^{\eta }\rangle$. The left eigenvectors can be obtained by writing $U_{\alpha }$ as $U_{\alpha }=V_{\alpha }\Lambda_{\alpha }V_{\alpha }^{-1}$, where $\Lambda_{\alpha }$ is diagonal and $\left\{|{\bar{\varepsilon }}_{\alpha n}^{\eta }\rangle \right\}$ correspond to the columns of ${\left(V_{\alpha }^{-1}\right)}^{†}$ [94]. Similar to the Q-matrix under the PBC, $Q_{\alpha }$ here can be viewed as an effective Hamiltonian of the Floquet operator $U_{\alpha }$, whose eigenvalues are set to 1 (−1) if the corresponding quasienergies of $U_{\alpha }$ satisfying Re(εnη)>0 [Re(εnη)<0]. With the help of ${\overset{˘}{w}}_{1}$ and ${\overset{˘}{w}}_{2}$ in Equation (23), we can construct another pair of topological invariants [84]:

(24) $${\overset{˘}{w}}_{0}=\frac{{\overset{˘}{w}}_{1}+{\overset{˘}{w}}_{2}}{2},\;{\overset{˘}{w}}_{\pi }=\frac{{\overset{˘}{w}}_{1}-{\overset{˘}{w}}_{2}}{2}.$$

In a fixed time frame α, previous studies have shown that ${\overset{˘}{w}}_{\alpha }=w_{\alpha }$ [84]. Therefore, we find the following bulk-edge correspondence relations for 1D non-Hermitian Floquet systems in the extended CII symmetry class, i.e.,

(25) $$\left(\right|w_{0}\left|,\right|w_{\pi }\left|\right)=\left(\right|{\overset{˘}{w}}_{0}\left|,\right|{\overset{˘}{w}}_{\pi }\left|\right)=\left(2n_{0},2n_{\pi }\right).$$

Since the second equality holds also under the OBC, it can be employed to investigate the effect of impurity on non-Hermitian Floquet topological phases, and characterize the non-Hermitian Floquet Anderson insulators that may appear due to the interplay between drivings, non-Hermiticity, and disorder. These topics are beyond the scope of the current work, and will be explored in the future.

Despite edge states, the topological signatures of non-Hermitian Floquet phases can also be extracted from bulk dynamics, as will be discussed in the next section.

## 5. Dynamical Probe to the Topological Phases

The mean chiral displacement (MCD) refers to the time-averaged chiral displacement $S\hat{n}$ of a wavepacket in a lattice, where S is the sublattice symmetry operator and $\hat{n}$ is the position operator of the unit cell. The MCD was first introduced as a dynamical probe to the winding numbers of 1D topological insulators in the symmetry classes AIII and BDI [98], and later extended to Floquet systems [93,99,100], two-dimensional systems [101], many-body systems [102], systems in other symmetry classes [84], and recently also to non-Hermitian systems [50,51,53]. In the meantime, the MCD has also been measured experimentally in photonic [98,103] and cold atom [104,105] setups. In this section, we further generalize the MCD to non-Hermitian Floquet systems in the CII symmetry class, and employ it to dynamically characterize the topological phases found in the non-Hermitian PQTLL model.

For a non-Hermitian Floquet system with sublattice symmetry S, we define the MCD $C_{\alpha }$ as the stroboscopic long-time average of the chiral displacement operator $S\hat{n}$ in a given symmetric time frame α (=1,2), i.e.,

(26) $$C_{\alpha }=\lim_{M\to \infty }\frac{1}{M}\sum_{m=1}^{M}\langle \bar{\psi }\left(m\right)|S\hat{n}|\psi \left(m\right)\rangle ,$$

where *m* counts the number of driving periods, which has been set to 1 following our choice of units. |ψ(m)〉 and $|\bar{\psi }\left(m\right)\rangle$ are the initial states evolved over *m*’s driving periods in the right and left Hilbert spaces, respectively. The $C_{\alpha }$ defined in this way is generally a complex number for finite *M* due to the implemented biorthogonal average. However, we will show that the imaginary part of $C_{\alpha }$ tend to vanish in the long-time limit M→∞.

Taking the Fourier transform from the position to momentum representation, and choosing the initial state to uniformly fill the non-Hermitian quasienergy band (ℓ,η) (ℓ=1,2, η=±), we find the following form of MCD:

(27) $$\begin{array}{cc} C_{\alpha \ell }^{\eta } & =\lim_{M\to \infty }\frac{1}{M}\sum_{m=1}^{M}\int_{-\pi }^{\pi }\frac{dk}{2\pi }c_{\alpha \ell }^{\eta }\left(k\right), \end{array}$$

(28) $$\begin{array}{cc} c_{\alpha \ell }^{\eta }\left(k\right) & =\frac{\langle {\bar{\varepsilon }}_{\alpha \ell }^{\eta }\left(k\right)|{\bar{U}}_{\alpha }^{m†}\left(k\right)Si\partial_{k}U_{\alpha }^{m}\left(k\right)|\varepsilon_{\alpha \ell }^{\eta }\left(k\right)\rangle }{\langle {\bar{\varepsilon }}_{\alpha \ell }^{\eta }\left(k\right)|{\bar{U}}_{\alpha }^{m†}\left(k\right)U_{\alpha }^{m}\left(k\right)|\varepsilon_{\alpha \ell }^{\eta }\left(k\right)\rangle }. \end{array}$$

Here $|\varepsilon_{\alpha \ell }^{\eta }\left(k\right)\rangle$ ($\langle {\bar{\varepsilon }}_{\alpha \ell }^{\eta }\left(k\right)|$) is the right (left) quasienergy eigenvector, and the corresponding Floquet operators can be expressed in the biorthogonal basis as:

(29) $$\begin{array}{cc} U_{\alpha }\left(k\right)= & \sum_{\ell ,\eta }e^{-i\varepsilon_{\ell }^{\eta }\left(k\right)}|\varepsilon_{\alpha \ell }^{\eta }\left(k\right)\rangle \langle {\bar{\varepsilon }}_{\alpha \ell }^{\eta }\left(k\right)|, \end{array}$$

(30) $$\begin{array}{cc} {\bar{U}}_{\alpha }^{†}\left(k\right)= & \sum_{\ell ,\eta }e^{+i\varepsilon_{\ell }^{\eta *}\left(k\right)}|\varepsilon_{\alpha \ell }^{\eta }\left(k\right)\rangle \langle {\bar{\varepsilon }}_{\alpha \ell }^{\eta }\left(k\right)|. \end{array}$$

Note that in Equation (27), a normalization factor has been added to cancel the changing norm of the state during the nonunitary evolution. Inserting the identity in biorthogonal basis $I=\sum_{\ell ,\eta }|\varepsilon_{\alpha \ell }^{\eta }\left(k\right)\rangle \langle {\bar{\varepsilon }}_{\alpha \ell }^{\eta }\left(k\right)|$, and using the orthonormality between left and right eigenvectors $\langle {\bar{\varepsilon }}_{\alpha \ell }^{\eta }\left(k\right)|\varepsilon_{\alpha \ell^{\prime }}^{\eta^{\prime }}\left(k\right)\rangle =\delta_{\ell \ell^{\prime }}\delta_{\eta \eta^{\prime }}$, the denominator of $c_{\alpha \ell }^{\eta }\left(k\right)$ in Equation (28) can be simplified as:

(31) 〈ε¯αℓη(k)|U¯αm†(k)Uαm(k)|εαℓη(k)〉=e2Im[εℓη(k)]m,

where Im[εℓη(k)] yields the imaginary part of the quasienergy $\varepsilon_{\ell }^{\eta }\left(k\right)$. Similarly, the numerator of $c_{\alpha \ell }^{\eta }\left(k\right)$ can be expressed as:

(32) 〈ε¯αℓη(k)|U¯αm†(k)Si∂kUαm(k)|εαℓη(k)〉=e2Im[εℓη(k)]m〈ε¯αℓη(k)|S|i∂kεαℓη(k)〉−ei2Re[εℓη(k)]m〈ε¯αℓ−η(k)|S|i∂kεαℓ−η(k)〉,

where we have also used the fact $S|\varepsilon_{\alpha \ell }^{\eta }\left(k\right)\rangle \propto |\varepsilon_{\alpha \ell }^{-\eta }\left(k\right)\rangle$ to arrive at the second term on the right hand side of Equation (32). Plugging Equations (31) and (32) into Equation (28), we find the integrand $c_{\alpha \ell }^{\eta }\left(k\right)$ to be:

(33) $$c_{\alpha \ell }^{\eta }\left(k\right)=\langle {\bar{\varepsilon }}_{\alpha \ell }^{\eta }\left(k\right)|S|i\partial_{k}\varepsilon_{\alpha \ell }^{\eta }\left(k\right)\rangle -\langle {\bar{\varepsilon }}_{\alpha \ell }^{-\eta }\left(k\right)|S|i\partial_{k}\varepsilon_{\alpha \ell }^{-\eta }\left(k\right)\rangle e^{i2\varepsilon_{\ell }^{\eta }\left(k\right)m}.$$

The first term on the right hand side of Equation (33) will be related to the winding number of the system in the α’s time frame. If Im[εℓη(k)]>0, the second term on the right hand side of Equation (33) will vanish in general after taking the sum over *m* and the limit M→∞, as imposed in Equation (27). However, when Im[εℓη(k)]<0, the factor $e^{i2\varepsilon_{\ell }^{\eta }\left(k\right)m}$ will contribute an exponentially growing factor to $c_{\alpha \ell }^{\eta }\left(k\right)$ after the summation over *m*, making it diverge in the limit M→∞.

To remove the divergence, we introduce another pair of Floquet propagators for the right and left initial states with Im[εℓη(k)]<0, which are given by:

(34) $$\begin{array}{cc} {\overset{´}{U}}_{\alpha }\left(k\right)= & \sum_{\ell ,\eta }e^{+i\varepsilon_{\ell }^{\eta }\left(k\right)}|\varepsilon_{\alpha \ell }^{\eta }\left(k\right)\rangle \langle {\bar{\varepsilon }}_{\alpha \ell }^{\eta }\left(k\right)|=U_{\alpha }^{-1}\left(k\right), \end{array}$$

(35) $$\begin{array}{cc} {\overset{`}{U}}_{\alpha }^{†}\left(k\right)= & \sum_{\ell ,\eta }e^{-i\varepsilon_{\ell }^{\eta *}\left(k\right)}|\varepsilon_{\alpha \ell }^{\eta }\left(k\right)\rangle \langle {\bar{\varepsilon }}_{\alpha \ell }^{\eta }\left(k\right)|={\left[{\bar{U}}_{\alpha }^{†}\left(k\right)\right]}^{-1}. \end{array}$$

Comapring with Equation (29), it is clear that ${\overset{´}{U}}_{\alpha }\left(k\right)$ is just the inverse of Floquet operator $U_{\alpha }\left(k\right)$, which can be obtained by simply reversing the signs of all the system parameters in our model. Note that ${\overset{´}{U}}_{\alpha }\left(k\right)$ and ${\overset{`}{U}}_{\alpha }\left(k\right)$ correspond to the Floquet operators in the left and right Hilbert spaces, respectively. They have different expressions in the biorthogonal Floquet eigenbasis, and are therefore distinguished by different accents on their heads. With these considerations, we modify the definition of $C_{\alpha \ell }^{\eta }$ in Equation (27) to:

(36) Cαℓη=limM→∞1M∑m=1M∫−ππdk2π·cαℓη(k)Im[εℓη(k)]>0cˇαℓη(k)Im[εℓη(k)]<0,

where $c_{\alpha \ell }^{\eta }\left(k\right)$ is given by Equation (28), and ${\overset{ˇ}{c}}_{\alpha \ell }^{\eta }\left(k\right)$ takes the form:

(37) $${\overset{ˇ}{c}}_{\alpha \ell }^{\eta }\left(k\right)=\frac{\langle {\bar{\varepsilon }}_{\alpha \ell }^{\eta }\left(k\right)|{\overset{`}{U}}_{\alpha }^{m†}\left(k\right)Si\partial_{k}{\overset{´}{U}}_{\alpha }^{m}\left(k\right)|\varepsilon_{\alpha \ell }^{\eta }\left(k\right)\rangle }{\langle {\bar{\varepsilon }}_{\alpha \ell }^{\eta }\left(k\right)|{\overset{`}{U}}_{\alpha }^{m†}\left(k\right){\overset{´}{U}}_{\alpha }^{m}\left(k\right)|\varepsilon_{\alpha \ell }^{\eta }\left(k\right)\rangle }.$$

Following the steps in the derivations of Equations (31) and (32), we find the denominator and numerator of ${\overset{ˇ}{c}}_{\alpha \ell }^{\eta }\left(k\right)$ to be:

(38) 〈ε˜αℓη(k)|U`αm†(k)U´αm(k)|εαℓη(k)〉=e−2Im[εℓη(k)]m,

(39) 〈ε¯αℓη(k)|U`αm†(k)Si∂kU´αm(k)|εαℓη(k)〉=e−2Im[εℓη(k)]m〈ε¯αℓη(k)|S|i∂kεαℓη(k)〉−e−i2Re[εℓη(k)]m〈ε¯αℓ−η(k)|S|i∂kεαℓ−η(k)〉.

Plugging them into Equation (37), we further obtain:

(40) $${\overset{ˇ}{c}}_{\alpha \ell }^{\eta }\left(k\right)=\langle {\bar{\varepsilon }}_{\alpha \ell }^{\eta }\left(k\right)|S|i\partial_{k}\varepsilon_{\alpha \ell }^{\eta }\left(k\right)\rangle -\langle {\bar{\varepsilon }}_{\alpha \ell }^{-\eta }\left(k\right)|S|i\partial_{k}\varepsilon_{\alpha \ell }^{-\eta }\left(k\right)\rangle e^{-i2\varepsilon_{\ell }^{\eta }\left(k\right)m}.$$

It is clear that under the condition Im[εℓη(k)]<0, the second term on the RHS of Equation (40) will in general vanish under the summation and long-time average $\lim_{M\to \infty }\frac{1}{M}\sum_{m}$, as imposed in Equation (36).

Next, we extend the initial state of our system to an incoherent summation of all uniformly filled Floquet bands (ℓ,η), which is equivalent to an “infinite-temperature” state at each quasimomentum *k*. In the lattice representation, such an initial state corresponds to the uniform population of all the four internal degrees of freedom (spins and sublattices) in the central unit cell of the ladder, which is relatively easy to prepare. For such an initial state, the MCD becomes $C_{\alpha }=\sum_{\ell ,\eta }C_{\alpha \ell }^{\eta }$. With the help of Equations (33), (36) and (40), it can be written more compactly as:

(41) $$C_{\alpha }=\sum_{\ell ,\eta }\int_{-\pi }^{\pi }\frac{dk}{2\pi }A_{\alpha \ell }^{\eta }\left(k\right)\left[1-\lim_{M\to \infty }\frac{1}{M}\frac{1-e^{i2s\varepsilon_{\ell }^{\eta }\left(k\right)M}}{e^{-i2s\varepsilon_{\ell }^{\eta }\left(k\right)}-1}\right],$$

where $A_{\alpha \ell }^{\eta }\left(k\right)\equiv \langle {\bar{\varepsilon }}_{\alpha \ell }^{\eta }\left(k\right)|S|i\partial_{k}\varepsilon_{\alpha \ell }^{\eta }\left(k\right)\rangle$, and s≡sgn{Im[εℓη(k)]} refers to the sign of Im[εℓη(k)]. It is not hard to see that the second term on the right hand side of Equation (41) will tend to vanish in the long-time limit M→∞, so long as $\varepsilon_{\ell }^{\eta }\left(k\right)=\pm \pi /2,\pm \pi$ have solutions only at a discrete set of *k*-points in the first Brillouin zone, which is the case for our system.

Finally, the relation between $C_{\alpha }$ and the winding number $w_{\alpha }$ in the α’s time frame can be established as follows. Inserting the expression of projector $Q_{\alpha }\left(k\right)$ in Equation (18) into the definition of $w_{\alpha }$ in Equation (17), and taking the trace in the biorthogonal basis, we find:

(42) $$w_{\alpha }=\int_{-\pi }^{\pi }\frac{dk}{4\pi }\sum_{\ell \ell^{\prime },\eta \eta^{\prime }}\eta \eta^{\prime }\langle {\bar{\varepsilon }}_{\alpha \ell }^{\eta }\left(k\right)|i\partial_{k}\left[|\varepsilon_{\alpha \ell^{\prime }}^{\eta^{\prime }}\left(k\right)\rangle \langle {\bar{\varepsilon }}_{\alpha \ell^{\prime }}^{\eta^{\prime }}\left(k\right)|\right]S|\varepsilon_{\alpha \ell }^{\eta }\left(k\right)\rangle .$$

Using again the orthonormality between left and right eigenvectors and the fact $S|\varepsilon_{\alpha \ell }^{\eta }\left(k\right)\rangle \propto |\varepsilon_{\alpha \ell }^{-\eta }\left(k\right)\rangle$, the expression for $w_{\alpha }$ can be simplified to:

(43) $$w_{\alpha }=\sum_{\ell ,\eta }\int_{-\pi }^{\pi }\frac{dk}{2\pi i}\langle {\bar{\varepsilon }}_{\alpha \ell }^{\eta }\left(k\right)|S|\partial_{k}\varepsilon_{\alpha \ell }^{\eta }\left(k\right)\rangle .$$

Comparing Equation (43) with Equation (41), we find the relation between the long-time averaged MCD $C_{\alpha }$ and winding number $w_{\alpha }$ as:

(44) $$w_{\alpha }=-C_{\alpha },\;\alpha =1,2.$$

Furthermore, with the help of the relations between $\left(w_{1},w_{2}\right)$ and the topological invariants $\left(w_{0},w_{\pi }\right)$ in Equation (19), we arrive at the relations between the MCDs and the topological winding numbers of 1D non-Hermitian Floquet systems in the CII symmetry class, i.e.,

(45) $$w_{0}=C_{0}\equiv -\frac{C_{1}+C_{2}}{2},\;w_{\pi }=C_{\pi }\equiv -\frac{C_{1}-C_{2}}{2}.$$

These relations establish a connection between the topology and dynamics of the non-Hermitian Floquet states in the system. They also provide us with a powerful way to probe the non-Hermitian Floquet topological phases in the CII symmetry class by measuring the MCDs experimentally in a pair of symmetric time frames.

**Figure 5:**
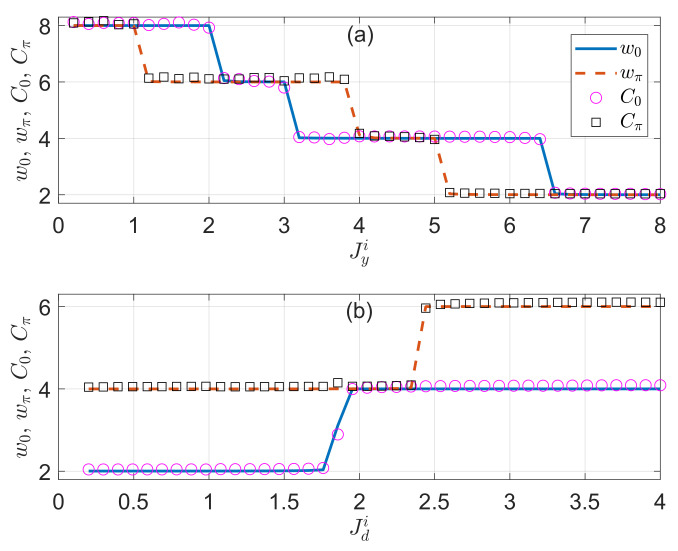
The topological winding numbers $w_{0}$ (blue solid lines), $w_{\pi }$ (red dashed lines), mean chiral displacements (MCDs) $C_{0}=-\frac{C_{1}+C_{2}}{2}$ (magenta circles), and $C_{\pi }=-\frac{C_{1}-C_{2}}{2}$ (black squares) versus the imaginary parts of vertical and diagonal hopping amplitudes $J_{y}^{i}$ and $J_{d}^{i}$ of the non-Hermitian PQTLL model in panels (**a**,**b**), respectively. The other system parameters are chosen as $\left(J_{x},J_{y}^{r},J_{d},V\right)=\left(0.5\pi ,1.5\pi ,4\pi ,0.1\pi \right)$ for panel (**a**) and $\left(J_{x},J_{y},J_{d}^{r},V\right)=\left(0.5\pi ,0.6\pi +6i,4\pi ,0.1\pi \right)$ for panel (**b**). The MCDs are averaged over M=20 driving periods for the results in both panels.

For completeness, we demonstrate the relations in Equation (45) by numerically simulating the dynamics. The results for two typical cases are presented in Figure 5a,b. In both panels, the time average is taken over M=20 driving periods, which is well within reach in current experiments. It is clear that the MCDs and topological winding numbers are consistent for all the non-Hermitian Floquet topological phases considered in the figure, and the small deviations are mainly originated from the finite-time effect. Furthermore, a quantized jump of the MCD is observed every time when the system passes through a topological phase transition point. Experimentally, the MCDs have been measured in both the cold atom [104,105] and photonic systems [98,103], in which non-Hermiticity and driving fields can also be implemented [1]. Furthermore, the MCDs may also be detected directly in momentum space with the help of a recently proposed setup certaining the nitrogen-vacancy-center in diamond [36]. Putting these together, we conclude that the MCD can indeed be employed as a dynamical probe to the topological phases and phase transitions in our non-Hermitian PQTLL model, and also in other 1D non-Hermitian Floquet systems in the CII symmetry class.

## 6. Conclusions

In this work, we introduced a periodically quenched two-leg ladder model subjecting to nonreciprocal inter-leg hoppings. The system belongs to an extended CII symmetry class in the non-Hermitian periodic table [9,11], which is further characterized by a pair of even-integer topological winding numbers $(w_{0},w_{\pi })\in 2Z\times 2Z$ due to the existence of time-periodic drivings. We established the topological phase diagrams of the model, and observed rich non-Hermitian Floquet topological phases with large winding numbers. In particular, Floquet phases carrying larger topological invariants can emerge in stronger non-Hermitian regimes thanks to the collaboration between drivings and non-Hermiticity. Under the open boundary condition, Floquet topological edge modes with zero and π quasienergies appear as fourfold degenerate quartets around the boundaries, whose exact numbers are determined by the bulk topological invariants $\left(w_{0},w_{\pi }\right)$. Besides the bulk-edge correspondence, we introduced the generalized mean chiral displacement as another probe to the topological features of our system dynamically, and showed that the MCDs in the long-time limit yield the topological invariants of one-dimensional non-Hermitian Floquet systems in the CII symmetry class. Our work not only uncovers a new type of topological phase originated from the interplay between drivings and non-Hermitian effects, but also paves the way for the dynamical characterization of non-Hermitian Floquet topological matter. In future work, it would be interesting to extend our findings to other symmetry classes, higher spatial dimensions, and superconducting systems. Furthermore, intriguing non-Hermitian Floquet phases and phenomena are expected to appear under the effects of disorder and many-body interactions, which certainly deserve thorough explorations.
